# The relative age effect shifts students’ choice of educational track even within a school system promoting equal opportunities

**DOI:** 10.3389/fpsyg.2022.1066264

**Published:** 2023-01-10

**Authors:** Geir Oterhals, Kari Elisabeth Bachmann, Annette Hessen Bjerke, Arve Vorland Pedersen

**Affiliations:** ^1^Faculty of Business Administration and Social Sciences, Molde University College, Molde, Norway; ^2^Department of Education, Faculty of Humanities and Education, Volda University College, Volda, Norway; ^3^Department of Psychology, School of Health Sciences, Education and Law, Kristiania University College, Oslo, Norway; ^4^Department of Primary and Secondary Teacher Education, Faculty of Education and International Studies, Oslo Metropolitan University, Oslo, Norway; ^5^Department of Neuromedicine and Movement Science, Faculty of Medicine and Health Sciences, Norwegian University of Science and Technology, Trondheim, Norway

**Keywords:** relative age effect, vocational track, academic track, gender, upper secondary school, Norway, tracking

## Abstract

In most education systems, the age of a given cohort of students spans up to 12 months, which creates a within-class age difference, or *relative age effect*, that tends to disadvantage younger students. Because birth month indeed correlates with academic performance, with poorer outcomes for students born later in the year, the effect can have lifelong consequences for students, whose academic performance justifies their acceptance into different educational tracks. Although past studies have identified the relative age effect in students’ choice of educational track in school systems in which students make such choices at the age of 10–14 years, we examined data from the Norwegian school system, in which education tracks are chosen at the age of 15–16 years. The dataset included the variables birth month, track choice, and gender, of all 28,231 pupils at the upper secondary school level in a school county in Norway. Birth month was compared between vocational and academic track choices and the results revealed a significant relative age effect on educational choices between academic and vocational tracks, such that younger students were significantly more likely to apply for vocational tracks. The effect was significantly stronger for boys compared to girls. This indicates that the choice of educational track may reflect students’ relative age, especially among boys, and hence, not be based on interests alone. Those findings have implications for actors involved in ensuring equity in education systems in Norway and elsewhere.

## 1. Introduction

When students have to choose between an academic track (AT) or a vocational track (VT), it is assumed that their choices are based on actual interests. However, students’ chosen educational trajectories seem to closely relate to their early academic performance (Trusty et al., 2000), which in turn is affected by the relative age effect (RAE)—that is, an advantage for students born early in the selection year when compared with their relatively younger peers. Numerous studies in education have shown the RAE in academic performance and its particular advantage for older students (e.g., Sprietsma, 2010; Ponzo and Scoppa, 2014; Aune et al., 2017; Peña, 2017; Solli, 2017; Olsen and Björnson, 2018; Bjerke et al., 2021). Because academic performance is influenced by the RAE, and because the choice of educational trajectory is based on early academic performance, it is likely that the RAE is also apparent in the choice of educational track.

In the case of early tracking at the age of 10–14 years, the RAE has indeed been found to relate to students’ choice of educational trajectory (Mühlenweg and Puhani, 2010; Jürges and Schneider, 2011; Ponzo and Scoppa, 2014; Schneeweis and Zweimüller, 2014; Hurwitz et al., 2015; Peña, 2020). However, to facilitate equity, the Organisation for Economic Co-Operation and Development (OECD, 2012) recommends tracking at a later stage. Some countries, including the Nordic countries, practise late tracking, meaning that all students follow the same path throughout compulsory education and do not choose between AT and VT until the age of 15–16 years (OECD, 2012; Fasting, 2013). In this paper, we analyse whether the RAE is also apparent in educational choices in the Norwegian school system, in which tracking is performed relatively late. In Norway, one statutory goal is to afford all students equal opportunities to learn and develop independently of their preconditions (Ministry of Education and Research, 2006). Equal opportunities are sought to be assured *via* a public school system with a very limited number of private schools. However, the growing body of research on the RAE in the Norwegian education system indicates that students are not given equal opportunities, partly due to their date of birth, among other factors. For instance, Solli (2017) found in a sample of students who completed secondary school from 1992 to 2003 that younger students had a relative age disadvantage in grade point average (GPA) and that the effect was stronger for students of lower socio-economic status. That same year, Aune et al. (2017) found a relative age advantage for older students in their marks in physical education in both lower and upper secondary school. In a more recent study, Bjerke et al. (2021) analysed data from Trends in International Mathematics and Science Study (TIMSS) in 2015, which in that particular year was conducted across four cohorts (i.e., Grades 4, 5, 8, and 9). Their analyses revealed significant correlations between birth month and performance in mathematics among students in Grades 4, 5, and 8 (Bjerke et al., 2021).

In the same vein, Olsen and Björnson (2018) found that the RAE is evident in the Programme for International Student Assessment (PISA) datasets and similar in size for performance in reading, science, and mathematics in Norway. Those results partly confirm a finding of Sprietsma (2010), who identified the RAE in PISA data on performance in reading and mathematics at age 15 years in 16 countries, including Norway. Strøm (2004) came to the same conclusion for reading tests in the PISA study measured at the end of compulsory school (i.e., age 15–16 years). Relative age differences have also been identified in national test scores for arithmetic skills (Aune et al., 2018) and reading skills (Vestheim et al., 2019) in Grades 5, 8, and 9 in Norway. Students born in January had up to 15% greater average scores than their peers born in December of the same year, with the greatest differences in Grade 5 (Aune et al., 2018). The same researchers (Aune et al., 2017, 2018; Vestheim et al., 2019) also found differences in relation to gender. In marks in physical education (Aune et al., 2017) and in scores on the national test of numeracy, boys born early in the year were overrepresented among high-performing students (Aune et al., 2018), whereas Vestheim et al. (2019) found that girls were overrepresented among high-performing students in reading literacy in Grades 5 and 9.

Studies have suggested that psychological components within the RAE maintain the effect over time, even after mental and physical maturation has levelled off (Fenzel, 1992; Thompson et al., 2004; Ponzo and Scoppa, 2014; Liu and Li, 2016). On the one hand, it is conceivable that younger students must work harder to keep pace with older students in their class and, over time, may thus outperform the ones born earlier in the year, thereby causing an inverse RAE, as has been observed in sports (Gibbs et al., 2012; Fumarco et al., 2017; Peña, 2017). On the other hand, negative experiences related to the assessment of school performance can lower self-esteem (Thompson et al., 2004) and weaken self-efficacy related to school performance at early school ages among the younger students (i.e., due to being compared with older students) and thus persist in a downward spiral throughout the educational process (Thompson et al., 2004; Liu and Li, 2016).

That downward spiral, or self-fulfilling prophecy (Rosenthal and Jacobson, 1968; Bjerke et al., 2021), may relate to findings showing that teachers perceive older students to be more professionally skilled (Jürges and Schneider, 2011; Baker et al., 2015) and provide more academic support and encouragement to students who are expected to perform well (Jussim and Harber, 2005). Researchers have also found that older students build better relationships with their teachers, which contributes to their greater self-confidence and stronger self-efficacy (Usher et al., 2019). Those outcomes, in turn, may be associated with the Pygmalion effect, which basically holds that the greater the expectations placed on a student, the greater results they will attain (Rosenthal and Jacobson, 1968). It is established that some teachers do form and communicate differential expectations to their students and that some students internalise these expectations in ways that manifest in their actual performance (Good et al., 2018).

Knowledge about when or whether the RAE mitigates in strength is important because a student’s birth month might influence their choice of educational track. Research has indicated that the RAE weakens as age and grade level increase (Aune et al., 2018; Bjerke et al., 2021). Those dynamics are somewhat unsurprising, given that maturational differences are levelled out by age. Even so, despite several studies on academic skills showing that the RAE is persistent (Strøm, 2004; Ponzo and Scoppa, 2014), there seem to be discrepancies between studies concerning at what age the RAE is attenuated. Whereas some studies have showed that the RAE is strongest during puberty and present in different areas many years afterwards (Peña, 2020), other studies have shown that the RAE on performance persists up to 14–16 years of age before becoming null (Sprietsma, 2010; Ponzo and Scoppa, 2014; Schneeweis and Zweimüller, 2014; Solli, 2017; Olsen and Björnson, 2018; Peña, 2020). In fact, Solli (2017) identified RAE in Norway from secondary school into adulthood, with a relative age disadvantage especially for boys, in rates of completion of high school, enrolment in college and earnings at the age of 30 years.

In the body of literature reporting on students at the academic milestone of choosing an educational programme in upper secondary school, Peña (2020) has shown that relatively older students both aim for and are more likely to end up in higher-quality schools. Moreover, Hurwitz et al. (2015) found that relatively younger students are less likely to take advanced placement exams, to more often sign up for high school programmes of shorter duration and to be less likely to obtain a bachelor’s degree 4 years after graduating from high school. In the United Kingdom, Crawford et al. (2013) found that the relatively younger students more often choose vocational subjects and are less represented in high-ranking research universities at the age of 19 years. Meanwhile, in Italy, Ponzo and Scoppa (2014) found that relatively older secondary school students are more likely to be tracked into academic schools than vocational schools. Even though some research (Sprietsma, 2010; Ponzo and Scoppa, 2014; Schneeweis and Zweimüller, 2014; Solli, 2017; Olsen and Björnson, 2018; Peña, 2020) has revealed that the RAE decreases as age and grade level increase, those studies have also been consistent in their findings that the effect relates to students’ performance and attributions in lower and upper secondary school as well.

Whether the RAE impacts educational choices seems to relate to each country’s education system, which differ in how they organise secondary education. Whereas some countries emphasise a comprehensive model of education and, by extension, practise late student tracking, other systems track students into separate groups based on their abilities or else introduce different pathways at a rather early age (Terrin and Triventi, 2022). In Austria, for example, the school system is arranged such that students (and their parents) have to choose between two fields of study at the age of 10 years (Schneeweis and Zweimüller, 2014). Students can choose either a lower secondary school that includes basic general education with preparation for vocational subjects or a more advanced academic education. Later, the final choice between vocational subjects and a more academic trajectory is made at the age of 14 years. In Austria, Schneeweis and Zweimüller (2014) found a strong RAE in the choice of education, such that students born late in the year more often chose a vocational-preparatory field of study, an effect that was evident at both 10 and 14 years of age. Germany has a similar system that involves choosing an AT or non-AT at the age of 10 years. In that context, Mühlenweg and Puhani (2010) found that the relatively younger students were significantly less likely to end up on an AT. Added to that, Jürges and Schneider (2011) found that relatively younger students in Germany were less likely to both be recommended to and enrolled in an AT, which also was regarded as the most attractive track in terms of outcomes later in life. In general, education systems with early tracking seem detrimental, especially for students who perform poorly from an early age, because many do not have sufficient time to compensate for maturational differences before choosing their programme of study (Ozer and Perc, 2020).

All those findings should not be interpreted to mean that students’ birth month alone predicts their educational choices, for several other factors are at play. For instance, it seems that ability-based grouping in primary school largely predicts future enrolment in higher education (Burger, 2021). In fact, 64% of the students in Switzerland on a high track in lower secondary school proceeded to an AT in upper secondary school and 70% of them proceeded to university. By contrast, 84% of the students on a low track in lower secondary school proceeded to vocational education, but only 4% of them proceeded to university (Burger, 2021). Also, in Switzerland, Helbling et al. (2021) were able to predict enrolment in academic or vocational tracks based on variables such as socio-economic status, cognitive abilities and motivation, with correct classifications increasing as students got closer to the end of elementary school. However, the RAE was not part of those authors’ prediction model.

In a Danish study, it was shown that upper secondary school tracking affected labour market outcomes and contributed in creating intergenerational inequality (Birkelund et al., 2021). Since the choice of educational track to such a great extent determines future labour market outcomes, it is of uttermost importance that the choices can be made based on students’ interests, and that the choice is not influenced by other factors like for instance the RAE.

Previous studies have shown that early tracking creates inequality among students, with disadvantages for ones born later in the academic year. In our study, we sought to identify whether students in Norway born late in the year more often prioritise vocational programmes in their application processes than their peers born early in the year. That potential dynamic is of special interest because students in Norway follow the same educational path until they turn 16 years old, which is generally at a later stage than reported in the mentioned literature on educational tracking. In Norway, after finishing compulsory school (i.e., lower secondary), students can choose which field of education they want to pursue, which is primarily a choice between different vocational studies and academic preparation.

Drawing on the literature showing that the RAE provides the relatively younger students with a weaker starting point at the end of lower secondary school, in our study we asked:

To what extent, if any, do relative age differences impact students’ choice of programme of study in upper secondary school when they are 16 years old?

## 2. Materials and methods

Anonymised registry data from the applications of students applying to upper secondary school were made accessible to us by the administration in one county municipality in Norway. Data included the birth date, gender and choice of programme of study on the application date (i.e., 1 March, in the same calendar year as entry into upper secondary school) for 28,231 students from 2012 to 2020. The sample included all applicants in the county who met the inclusion criterion of (1) being 15–16 years old at the time of application (i.e., grade repetition is almost absent in Norway’s school system; OECD, 2012), (2) having an ordinary Norwegian national identity number including day and month of birth and (3) who prioritised certain programmes of study in their application, as is required. Because the students were allowed to apply for whichever school they wanted, for some students, distance from home to the institutions might had influenced their list of prioritised schools. However, that effect probably applied to only a minor proportion of the sample and did not affect the primary results. In the data from the applications, only each student’s top choice of programme of study was registered, namely either as an AT or VT. The distribution of applicants across the 9-year period appears in Table 1.

**Table 1:** Number of applicants from the academic years 2012–2013 to 2020–2021 for the 9  years studied, beginning at the March application date.

	Girls AT	Boys AT	Girls VT	Boys VT	Total
2012–2013	785	592	630	985	2,992
2013–2014	865	606	585	967	3,020
2014–2015	904	598	552	989	3,043
2015–2016	959	618	646	1,043	3,266
2016–2017	1,028	735	521	951	3,235
2017–2018	1,074	722	521	908	3,225
2018–2019	979	611	525	913	3,028
2019–2020	958	692	555	957	3,162
2020–2021	953	678	607	992	3,260
Total	**8,535**	**5,852**	**5,139**	**8,705**	**28,231**

AT, academic track; VT, vocational track.

The following hypotheses were tested:

*Hypothesis 1*: A correlation exists between birth month and choice of programme of study for both girls and boys on the date of application.

*Hypothesis 2*: The difference in the portion of students born early and students born late who choose a VT is greater for boys than for girls.

Our categorical data with two different outcomes, VT or AT, were treated as shares of students. To test Hypothesis 1, we used linear bivariate models, with the share of students choosing VT, *Y*_i_, as a function of *BirthMonth*_i_ for share *i*—that is, *Y*_i_ = *b*_0_ + *b*_1_ · *BirthMonth*_i_. If *b*_1_ was positive, the trend was that later months correlated with larger shares of VT applicants. Three separate regressions were run: one for the whole dataset, one for girls and one for boys. Fisher’s *z* test for differences between correlation coefficients in independent samples was used to test Hypothesis 2 (Asuero et al., 2006).

## 3. Results

**Figure 1:**
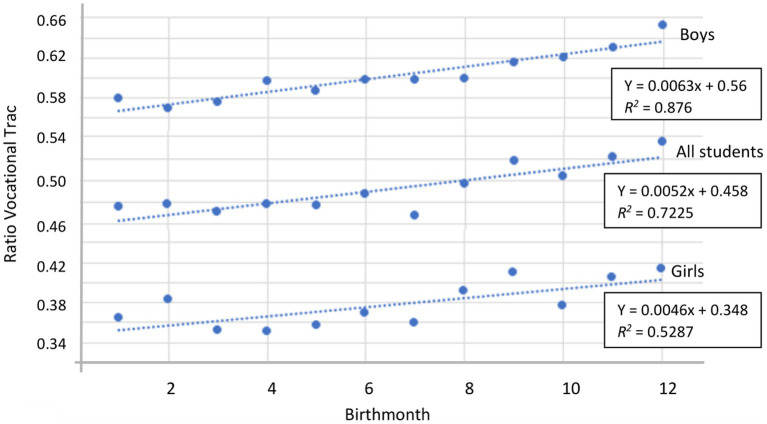
Regression lines with *R*^2^ for boys (upper line), all students (middle line) and girls (bottom line) showing ratio of students choosing vocational tracks (*y*-axis) vs. birth month (*x*-axis). Ratios exceeding 0.5 indicate more students applying for vocational tracks.

Significant correlations at the 0.01 level were found between birth month and choice of programme of study for all students (*b*_1_ = 0.0052, *r = 0*.85 [*z =* 5.69]), for girls (*b*_1_ = 0.0046, *r = 0*.73 [*z =* 2.95]) and for boys (*b*_1_ = 0.0063, *r = 0*.94 [*z =* 4.99]), which supported Hypothesis 1. The regression lines in Figure 1 indicate that most students born between January and July chose ATs, whereas those born in August or later more often chose VTs.

Hypothesis 2, asserting that the RAE would be stronger among boys than among girls, was also supported. Fisher’s *z* test for differences between correlation coefficients in independent samples (i.e., comparison of correlations from independent samples) yielded a result of *z =* 65.56, which was significant at the 0.01 level.

## 4. Discussion

The data that we analysed revealed the RAE in the choice of educational track in upper secondary school among 15–16-year-old students in Norway. A higher proportion of students born relatively early in the calendar year chose an AT, whereas those born later in the year more often chose VTs. The trend is clearly visible in Figure 1, which shows that students born in any of month through August, albeit with only a marginal difference in August, more often chose an AT, whereas those born in September through December more often chose VTs. Thus, the data show that the RAE affects students’ educational choices, even at the age of 16 years, within Norway’s highly equity-focused education system. That result is interesting considering that Black et al. (2011), using administrative data from Norway, reported that starting school younger has little effect on educational attainment.

In our study, the RAE was larger for boys than for girls, which may be because puberty commences later among boys than among girls and affects boys born late in the year particularly harder. Later-born boys are liable to experience a double whammy relative to girls in general and even more so compared with girls born early in the calendar year and thus effectively become up to several years behind in their development (Musch and Grondin, 2001). Thus, both their gender and their birthdate would be associated with a developmental lag compared with their peers. As discussed in the introduction of this paper, boys are known to be overrepresented in a host of populations with developmental problems (Root et al., 2019), as well as to receive poorer marks than girls (e.g., Statistics Norway, 2009; Aune et al., 2017, 2018; Vestheim et al., 2019). The same holds for children born late in the year, as has been demonstrated in a plethora of studies on the RAE. The fact that boys are hit so hard due to lagging in development contributes to the explanation of gender differences in school. Teachers might either be unaware of the effect or aware of it but unable to compensate for the differences, thereby inducing effects such as self-fulfilling prophecies and or the Pygmalion effect (Rosenthal and Jacobson, 1968).

The choice of educational track, of course, is not due to age *per se* but most likely because later-born students tend to have lower GPAs. Again, that outcome might be due to those students’ being less developed than their earlier-born peers (Dalen et al., 2017). It is also possible to assume that low marks and other negative experiences related to feedback on school performance will have a negative effect on students’ socioemotional development over time, including their self-esteem (Thompson et al., 2004) and self-efficacy, which results in a downward spiral throughout the educational process (Liu and Li, 2016) that influences the choice of educational track. In our study, we did not include data purporting differences in GPA between earlier- and later-born students; even so, it is known that the RAE exists in GPA to the advantage of older students (Solli, 2017). The RAE in our study, as in studies on the RAE in general, was used as a proxy for relative development. As a proxy, however, the RAE is as good as any, since on average, every anthropometric and physiological variable during adolescence is almost linearly associated with age (Júlíusson et al., 2009), meaning that relative age is a good indicator of relative development. It has even been argued that the RAE should instead be labelled the “relative development effect” (Bjerke et al., 2020). Of course, large differences exist between individuals, as do large systematic differences between groups of individuals, as our results show.

The data that we analysed show that the RAE is strong, at least until 16 years of age, which would support arguments that academic tracking should occur rather late (OECD, 2012) when the goal is to give students equal opportunities independent of their preconditions. Ozer and Perc (2020) have argued that tracking for different programmes should be done as late as possible. Such recommendations are also supported by findings that the RAE substantially affects the choice of track in school systems with early tracking ages (Mühlenweg and Puhani, 2010; Schneeweis and Zweimüller, 2014) and findings by Terrin and Triventi (2022) that late tracking can reduce inequality in educational opportunities. At the same time, early tracking could arguably benefit students if it occurs when the RAE has not been in effect for especially long—thus, neither have secondary effects (e.g., the Pygmalion effect) and thus does not affect GPA as much. That choice, however, would probably be made by the parents and not so much by informed, competent students.

The finding of the RAE’s considerable influence on the choice of educational track in the Norwegian school system is quite puzzling given the system’s goal of equal opportunities (Fasting, 2013; Haug, 2017) and the frequent use of adapted education to ensure equal opportunities for students. That the highly homogeneous Norwegian system is so affected by the RAE indicates that the effect is extremely strong and may have crept under the radar among variables known to induce differences between students. Knowing how pervasively the RAE affects other variables in the school system (e.g., Sprietsma, 2010; Ponzo and Scoppa, 2014; Aune et al., 2017; Peña, 2017; Solli, 2017; Olsen and Björnson, 2018; Bjerke et al., 2021), it should perhaps not come as a surprise that school performance may be affected as well.

To further investigate the underlying mechanisms behind students’ choices between VTs and ATs in upper secondary education in Norway, future studies should distinguish between different educational programmes within VTs.

A possible limitation of our study is that it included only a single county in Norway. However, because the Norwegian school system is among the most homogeneous in the world (see Zhang et al., 2015), the results would likely be similar in other regions. According to Zhang et al. (2015), Norway, together with Denmark, has the lowest between-school variance in PISA test scores among all participating nations, and, per the OECD (2013), Norway had one of the smallest variations in performance between schools across OECD member countries on the PISA 2009. Furthermore, Norway is one of the world highest-ranked education systems when it comes to combining equal opportunities with quality. According to the OECD (2012), reasons for the school system’s uniformity include that school assignments are done by catchment area instead of school choice, there is no grade repetition, and there are strict restrictions on grouping by ability as set out in the Education Act.

Nevertheless, any difference between regions would be of interest and motivate further study. In our study, however, because we did not examine the choice of educational track *per se*, our findings do not capture the pure choice of AT versus VT. After all, a specific region could differ from the remainder of a country due to geographic or social factors in that children in typical rural areas more often choose VTs. Even so, our study revealed a difference in the choice of AT between students born early versus later in the same year and living in the same region.

Last, even though our findings are based on associations, they do not indicate the direction of those associations. Thus, we cannot know for certain that relative development (represented by the proxy RAE) causes lower GPA and consequently the choice of track. However, since choice of AT obviously cannot determine when in the calendar year a student is born, the only other possibility is other variables affecting both the GPA and academic preference, or interests, of students.

## 5. Conclusion

The present study shows that the RAE is present and strong in the choice of educational track, even in the Norwegian education system which is promoting and working towards providing equal opportunities for all students. Students born in the first months of the year up to and including July, more often chose academic tracks, while the trend was opposite among students born in September to December, who more often chose vocational tracks. It is not at all logical that such a shift occurs because of interest, and that those born relatively late in the year should be more interested in vocational studies, while those born early in the year are more interested in academic studies. The effect is particularly strong among boys, who are already as a group disadvantaged compared with girls. Boys born late in the year, thus, experiences a double whammy, being disadvantaged both relative to girls and to earlier born boys. The findings are of crucial importance as upper secondary school tracking might affect later labour market outcomes and reproduce intergenerational inequalities. The findings have implications for actors involved in ensuring equity in education systems across countries, and in Norway specifically.

## Data availability statement

The raw data supporting the conclusions of this article will be made available by the authors, without undue reservation.

## Author contributions

GO, AP, and KB conceived the idea and designed the study. AB analysed the data received from Møre and Romsdal county municipality. GO wrote the first draft of the manuscript. All authors contributed to the article and approved the submitted version.

## Conflict of interest

The authors declare that the research was conducted in the absence of any commercial or financial relationships that could be construed as a potential conflict of interest.

## Publisher’s note

All claims expressed in this article are solely those of the authors and do not necessarily represent those of their affiliated organizations, or those of the publisher, the editors and the reviewers. Any product that may be evaluated in this article, or claim that may be made by its manufacturer, is not guaranteed or endorsed by the publisher.
